# Comprehensive Analysis of HPV16 Integration in OSCC Reveals No Significant Impact of Physical Status on Viral Oncogene and Virally Disrupted Human Gene Expression

**DOI:** 10.1371/journal.pone.0088718

**Published:** 2014-02-24

**Authors:** Nadine C. Olthof, Ernst-Jan M. Speel, Jutta Kolligs, Annick Haesevoets, Mieke Henfling, Frans C. S. Ramaekers, Simon F. Preuss, Uta Drebber, Ulrike Wieland, Steffi Silling, Wan L. Lam, Emily A. Vucic, Bernd Kremer, Jens-P. Klussmann, Christian U. Huebbers

**Affiliations:** 1 Department of Otorhinolaryngology and Head and Neck Surgery, GROW - School for Oncology and Developmental Biology, Maastricht University Medical Centre, Maastricht, the Netherlands; 2 Department of Molecular Cell Biology, GROW - School for Oncology and Developmental Biology, Maastricht University Medical Centre, Maastricht, the Netherlands; 3 Department of Pathology, GROW - School for Oncology and Developmental Biology, Maastricht University Medical Centre, Maastricht, the Netherlands; 4 Jean-Uhrmacher-Institute for Otorhinolaryngological Research, University of Cologne, Cologne, Germany; 5 Department of Otorhinolaryngology, Head and Neck Surgery, University Hospital of Cologne, Cologne, Germany; 6 Institute for Pathology, University Hospital of Cologne, Cologne, Germany; 7 Institute of Virology, National Reference Centre for Papilloma- and Polyomaviruses, University Hospital of Cologne, Cologne, Germany; 8 Department of Integrative Oncology, British Columbia Cancer Research Centre, Vancouver, Canada; 9 Department of Otorhinolaryngology, Head and Neck Surgery, University Hospital of Giessen, Giessen, Germany; Kagoshima University Graduate School of Medical and Dental Sciences, Japan

## Abstract

Infection with high-risk human papillomavirus (HPV) type 16 is an independent risk factor for the development of oropharyngeal squamous cell carcinomas (OSCC). However, it is unclear whether viral integration is an essential hallmark in the carcinogenic process of OSCC and whether HPV integration correlates with the level of viral gene transcription and influences the expression of disrupted host genes. We analyzed 75 patients with OSCC. HPV16-positivity was proven by p16^INK4A^ immunohistochemistry, PCR and FISH. Viral integration was examined using DIPS- as well as APOT-PCR. Viral *E2*, *E6* and *E7* gene expression levels were quantified by quantitative reverse transcriptase (RT-q)PCR. Expression levels of 7 human genes disrupted by the virus were extracted from mRNA expression profiling data of 32 OSCCs. Viral copy numbers were assessed by qPCR in 73 tumors. We identified 37 HPV16-human fusion products indicating viral integration in 29 (39%) OSCC. In the remaining tumors (61%) only episome-derived PCR products were detected. When comparing OSCC with or without an integration-derived fusion product, we did not find significant differences in the mean RNA expression of viral genes *E2*, *E6* and *E7* or the viral copy numbers per cell, nor did the RNA expression of the HPV-disrupted genes differ from either group of OSCC. In conclusion, our data do not support the hypothesis that integration affects the levels of viral and/or HPV-disrupted human gene transcripts. Thus constitutive, rather than a high level, of expression of oncogene transcripts appears to be required in HPV-related OSCC.

## Introduction

Approximately 600.000 new cases of head and neck squamous cell carcinoma (HNSCC) have been estimated to occur worldwide in 2011, ranking them in sixth position of all carcinomas [1]–[3]. Risk factors for the development of HNSCC include environmental factors, excessive tobacco and alcohol use, as well as human papillomavirus (HPV) infections. Particularly oropharyngeal squamous cell carcinomas (OSCC) are associated with HPV16 [4]. This group of carcinomas shows clinicopathological and molecular characteristics that differ from alcohol- and tobacco-induced carcinomas [4]–[6]. Studies that have assessed the prevalence of HPV-induced OSCC report frequencies ranging from 20% to up to 90% [5],[7]–[9].

Although integration of the viral DNA into the host genome is not part of the normal viral life cycle, studies in anogenital carcinomas have shown a significant correlation between integration and progression of dysplastic lesions to invasive carcinomas [10],[11]. For example in uterine cervical carcinomas, it has been shown that oncogene transcripts indicating viral integration can be identified in 55% of HPV16 positive cases and 92% of the HPV18 positive cases, and that particularly for HPV16 the integration events have been found to occur already in cervical intraepithelial neoplasia (CIN) [11]. We recently also detected viral integration in head and neck oropharyngeal dysplasia adjacent to squamous cell carcinoma by FISH, however these dysplasia are a rare finding in the oropharynx [12].

Using Amplification of Papillomavirus Oncogene Transcripts PCR (APOT-PCR), so far only two studies report HPV16 integration in 2 out of 4 and 6 out of 9 tumors in HPV-DNA positive OSCC [13],[14].

However, there is controversy with respect to the relation between viral integration and viral gene expression. Integration of HPV DNA in uterine cervical squamous cell carcinomas (UCSCC) has been correlated to disruption of the viral regulatory gene *E2* [15],[16]. Studies in cell lines have shown that E2 represses the viral *E6* and *E7* expression [17]. In the uterine cervical cell line W12, integration of HPV was shown to result in higher levels of the oncogenes *E6/E7* and a selective growth advantage over cells harboring extrachromosomal HPV DNA [18]. This had led to the hypothesis that the levels of viral *E6* and *E7* transcripts are higher in lesions in which viral integration resulted in *E2* disruption, which is thought to lead to deregulation of cell cycle control [19]–[21].

On the other hand, a study in primary keratinocytes immortalized with HPV16 genomes has shown that disruption of the *E2* gene sequence upon viral integration does not result in increased expression of the viral *E6* and *E7* oncogenes [13]. In addition, a publication by Häfner et al. using APOT-PCR has shown no correlation between the integration state of the viral genome and the expression of the viral gene E6 in a collection of 55 HPV16-positive cervical carcinoma samples [22]. It would be interesting to examine viral physical status and *E2*, *E6* and *E7* gene expression in primary OSCC since this information is lacking.

Here we present the HPV16 integration status for a collection of 75 HPV16-DNA-positive and p16^INK4A^-positive OSCC using APOT- and Detection of Integrated Papillomavirus Sequences PCR (DIPS-PCR), and its relation to the level of gene expression for the viral genes *E2*, *E6* and *E7*, gene expression analysis of a number of human genes disrupted by viral integration and viral DNA-load.

## Materials and Methods

### Subjects and Material

Fresh frozen clinical OSCC samples from 75 patients treated at the Departments of Otorhinolaryngology and Head and Neck Surgery of the University Hospitals of Cologne and Maastricht between 1994 and 2009 were collected from the archives of the Departments of Pathology of both hospitals. Inclusion criteria were the availability of sufficient fresh frozen tumor tissue containing ≥70% tumor cells, high quality tumor DNA and RNA and HPV16 infection, as detected by HPV-specific PCR and FISH analysis [4],[5],[23],[24] and overexpression of the surrogate marker p16^INK4A^ as detected by immunohistochemistry [25],[26].

Patient age ranged from 44–83 years (median 62.7 years). Fifty-seven (76.0 %) patients were male and eighteen (24.0 %) were female.

### Ethics Statement

Patient material was used according to the code for proper secondary use of human tissue. The ethics committees of the Universities of Cologne and Maastricht medical faculties approved this study. Written, informed consent had been obtained from all patients.

### Amplification of Papillomavirus Oncogene Transcripts PCR (APOT-PCR)

Total RNA was extracted from five 10 µm-thick snap frozen tissue sections using the RNeasy mini kit (Qiagen, Hilden, Germany) and DNase treatment. RNA concentration and quality were determined by RNA StdSens Chips on a BioRad Experion system (BioRad, Munich, Germany). HPV oncogene transcripts were amplified as described before [27]. Briefly, reverse transcription was performed using 25 µM oligo-(dT)_17_ primer coupled to a linker sequence (dT)_17_-p3, 10 mM dNTPs each, 0.1 M DTT, 5× RT-buffer and SuperScript reverse transcriptase (Invitrogen, Karlsruhe, Germany) [28]. Quality of transcribed cDNA was determined by a standard *GAPDH* gene PCR (441 bp product). First-strand cDNAs containing viral oncogene sequences were subsequently amplified with semi-nested PCR using HPV-E7 specific 5′-primers and oligo(dT) and adaptor primers (3′). PCR products were separated on a 1.2% agarose gel (see Figure S1 for representative PCR results). Both bands typical for episomal and integration status were cut out, purified using the QIAGEN Gel extraction kit (QIAGEN, Hilden, Germany) and sequenced (GATC Biotech, Konstanz, Germany). Sequence results were analyzed using the BLASTN program and further mapped using map viewer (both NCBI) [29],[30].

### Detection of Integrated Papillomavirus Sequences PCR (DIPS-PCR)

Integrated papillomavirus sequences were detected using the Detection of Integrated Papillomavirus Sequences-PCR (DIPS-PCR) assay, as described earlier [31]. Briefly, genomic DNA was digested using the *Sau*3AI restriction enzyme and an enzyme-specific adapter was ligated to the restriction-digested DNA using T4 DNA ligase (Roche Diagnostics, Mannheim, Germany). Linear PCR was performed using 5 HPV16 specific forward primers in independent setups, all using the same specific adapter primer 1 (AP1). All independent PCRs were followed by individual exponential PCRs using further virus-specific forward primers and the AP1 reverse primer. PCR products were separated on a 1.2% agarose gel and products of interest were excised, purified, sequenced and analyzed as described before (see Figure S1 for representative PCR results).

### Gene Expression Analysis

#### mRNA Expression Profiling

Total RNA was collected from a subset of 32 samples, randomly selected from the 75 patients in this study. Samples were analysed using Agilent Whole Human Genome 4×44K Microarrays, which represent more than 41,000 unique human transcripts. Labelling and hybridizations were performed according to the manufacturer's instructions (Agilent Technologies). Hybridized arrays were scanned using an Axon GenePix 4000B or 4200A scanner. Microarray analysis was performed using GenePix Pro 6.0.1.25. For normalization processing, the median array intensity was calculated based on the background-subtracted intensity value for all spots excluding control type spots on the array. The background-subtracted intensity value of each spot was then divided by the median array intensity of each microarray.

In the 32 tumor samples, 6 samples showed fusion products that were located within 7 genes. Normalized expression data for these genes were collected for all 32 samples. Per gene the expression in the sample with integration in that gene was compared to samples with or without an identified fusion product. Graphs were made using Graph Pad Prism 5.

#### HPV16 Oncogene Expression by qPCR

**Figure 1:**
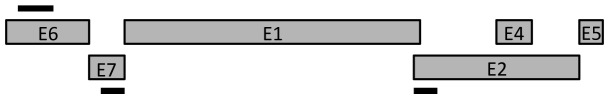
HPV16 genome showing the localization of the RT-qPCR-products obtained for *E2*, *E6* and *E7* viral oncogenes.

RNA isolated from 61 samples of which sufficient RNA was available after APOT analysis, was reverse transcribed using the iScript cDNA Synthesis Kit (BioRad Laboratories, Hercules, CA, USA). qPCR reactions were performed using SensiMix SYBR & Fluorescein (GC Biotech, Alphen a/d Rijn, the Netherlands). The following HPV-specific primers were used (see Figure 1): *E2* (87 bp product) forward primer 5′- TGATAGTACAGACCTACGTGACCATATAGA-3′ (Primer Express v2.0 (Applied Biosystems, Carlsbad, USA)) and reverse primer 5′- ATTACAAGGCCAGAGAAATGGG-3′ (Primer Express); *E6* (106 bp product) forward primer 5′-CAGTTATGCACAGAGCTGCAA-3′ [32] and reverse primer 5′- ATGACTTTGCTTTCGGGATT-3′ [32] ; *E7* (86 bp product) forward primer 5′-AGAGGAGGAGGATGAAATAGATGGT-3′and reverse primer 5′-CAATATTGTAACCTTTTGTTGCAAGTG-3′ (designed using Primer-BLAST (National Center for Biotechnology Information (NCBI)) [33]. Because of limited material, E7 expression analysis was performed on 45 samples. The detection of the housekeeping gene *Hypoxanthine Phosphoribosyltransferase* (HPRT) was used for normalization of mRNA levels: forward primer 5′-CACTGGCAAAACAATGCAGACT -3′ and reverse primer 5′-GTCTGGCTTATATCCAACACTTCGT -3. Cell lines SiHa and CaSki were used as positive controls. The HPV18-positive cell line HeLa was used as a control for HPV16 specificity and expression of viral genes did not yield values higher than the background signal.

### Fluorescence *In Situ* Hybridisation (FISH) for Co-localization of HPV16 and Human DNA Sequences

**Figure 2:**
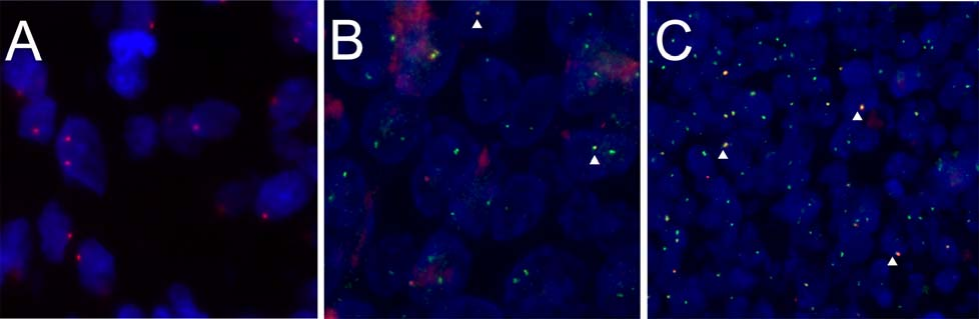
Representative examples of three different (Co-)FISH staining patterns: Representative sample of a HPV-positive FISH staining (A). Representative examples of co-hybridization using both a specific BAC-clone and the HPV16 probe (B–C). (B) BAC-clone RP11-731I19 (containing *BRE*), case 2, table S1. (C) BAC-clone RP11-299P2 (containing *BCL2*), case 21. Arrowheads indicate co-localization.

FISH on 4 µm thick tissue sections of formalin-fixed, paraffin-embedded tumour blocks was performed as described previously [5],[25],[34]. Briefly, sections were deparaffinised, pretreated with 85% formic acid/0.3% H_2_O_2_, 1 M NaSCN and 4 mg/ml pepsin, postfixed in 4% formaldehyde in PBS and dehydrated in an ascending ethanol series. For the co-localization experiments, we used biotin-labelled HPV16 probes (PanPath, Amsterdam, the Netherlands) together with digoxigenin-labelled BAC-clones containing human DNA sequences also identified in the viral-human fusion PCR products obtained by APOT/DIPS PCR. These BAC clones were grown according to the manufacturer's instructions (BACPAC Resources Centre, Childrens Hospital Oakland Research Institute, Oakland, USA). DNA was isolated using the Nucleobond BAC-100 kit (BioKé, Leiden, the Netherlands). Both probes were applied under a coverslip in a hybridization mixture containing 2 ng/µl HPV16 probe, 10 ng/µl BAC probe, 50% formamide, 2×SSC pH 7.0, 50× excess salmon sperm DNA (Sigma) and 10× excess human CoT DNA. Probes and tissue DNA were denatured simultaneously for 5 minutes at 80°C prior to hybridization overnight at 37°C in a humid chamber. After hybridization the preparations were washed stringently in 2× SSC+0.05% tween-20 at 42°C (2 times 5 min), 0.1× SSC at 61°C (2 times 5 min) and 4× SSC+0.05% tween-20 at RT. Biotin-labelled probe was detected using peroxidase-conjugated-avidine (Dako, Glostrup, Denmark; 1∶200 diluted in 4× SSC containing 5% non-fat dry milk; 30 minutes at 37°C) and biotin-conjugated goat-anti-avidine (Vector laboratories, Burlingame, CA; 1∶100 diluted in 4× SSC containing 5% non-fat dry milk; 30 minutes at 37°C). Thereafter, a tyramide signal amplification reaction was performed under a coverslip by applying 50 µl Cy3-labelled tyramide in PBS containing 0.1 M imidazole, pH 7.6 and 0.001% H_2_O_2_ for 10 minutes at 37°C. The digoxigenin-labelled probe was detected using mouse-anti-digoxigenin (Dako; 1∶200), followed by TRITC-conjugated rabbit-anti-mouse (Dako; 1∶1000) and finally TRITC-conjugated swine-anti-rabbit (Dako; 1∶100); incubated for 30 minutes at 37°C. Finally, slides were washed and dehydrated in an ascending ethanol series and mounted in Vectashield (Vector laboratories, Burlingame, CA) containing 4′,6-diamidino-2-phenylindole (DAPI; Sigma; 0.2 µg/µl). See Figure 2 for representative samples.

### Viral Load

Viral load of HPV16 was determined using real-time fluorescence PCR with type-specific primers and probes as described earlier [26]. Briefly, viral load was expressed as the number of HPV16 DNA copies per *β-globin*-gene copy. Gene copy numbers of *β-globin* were determined using the LightCycler-Control Kit DNA (Roche Molecular Biochemicals) according to the manufacturer's instructions as previously described [35]. Calculation of initial copy numbers in samples was performed by the LightCycler 480 software (Version 1.5) using a standard curve generated with exactly quantified HPV DNA standards (ten-fold dilution series of full length HPV16 plasmid) that were amplified in the same PCR run [35]–[37]. The analytical sensitivity of the assay was ten copies of HPV16 standard DNA. A negative control (water or DNA extracted from RTS3B cells that are negative for HPV) was included in each run and never yielded fluorescence signals above the background [35].

### Statistics

Differences in viral and human gene expression levels were analyzed using a 2-tailed Fisher's exact test after testing for equality of variances. A significance level of p<0.05 was chosen for all analyses. To test whether a single sample deviated from a group of samples, SPSS was used to identify outliers. This was defined as any value that lays more than 1.5 times the interquartile range below the first quartile in a Box-and-Whisker Plot from all samples, or more than 1.5 times the interquartile range above the third quartile. All calculations were performed using IBM SPSS Statistics 19.

## Results

### Detection of Viral Integration by DIPS- and APOT-PCR

Table S1 and Figure S2 summarize the integration sites of HPV16 into the genome of 29 of the 75 HPV16 DNA- and p16^INK4A^-positive OSCC (39%) as identified by DIPS- and APOT-PCR. Exclusively episomal PCR products were detected in the remaining 46 tumors (61%).

In the 29 tumors with viral integration a total of 37 fusion products were identified, of which 10 harbored cellular sequences corresponding to intergenic regions and 27 to known or predicted genes, including 12 tumor-related genes (*BCL2*, *BRE*, *EPHA7*, *FANCC* (2×), *HDAC2*, *INO80C*, *LEPREL1*, *SYNPO2*, *TP63*, *TRAF3*, *TUBD1*), 5 genes involved in deregulated tumor-related pathways (*ERC2*, *GARS*, *SLC7A1*, *SYN3*, *ZMAT4*), and 10 genes with no known role in tumorigenesis. All genes were verified using the ATLAS of genetics and cytogenetics in oncology and hematology Database [38] and the UniProtKB Database [39]. Figure S3 summarizes viral-cellular splicing observed in this study, including a new type D splicing not described before.

### Detection of Gene Expression

Subsequently, we analyzed whether HPV16 integration as detected by PCR, correlated with the level of expression of the disrupted gene. In addition, we determined whether integration correlated with the expression of the viral genes *E2*, *E6* and *E7*.

#### Expression of Genes Disrupted by HPV

**Figure 3:**
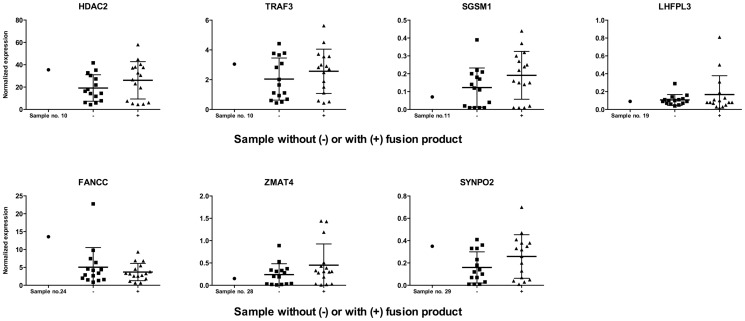
Expression intensities for genes affected by HPV integration. The expression of a gene, affected by HPV integration in one sample, is compared to the expression of that gene in samples where exclusively episomal PCR products could be detected using APOT- and/or DIPS-PCR, and to the expression in samples where fusion products could be identified. Bars: Mean with standard deviation.

We extracted the level of expression of HPV-disrupted genes from expression profiling data of a subset of 32 OSCC, in which HPV16 integrated within a gene in 6 out of 32 OSCC, one of which contained two different integration sites (sample 10, Table S1). For each gene, its expression was compared between the single sample with HPV integration in the affected gene, the group of samples showing exclusively episomal PCR products (n = 20) and the group of samples with fusion products harboring sequences derived from other chromosomal loci (n = 11) (Figure 3). In all cases there was no significant change in the mean mRNA expression levels of the HPV-disrupted genes between the subgroups with or without a fusion product. In the tumor with the HPV integration in the particular gene, the expression did not surpass the 1.5 interquartile range (IQR) of the group of samples with integration-derived fusion products, as calculated using SPSS, in 6 of the 7 genes. In the tumor with integration in the *FANCC* gene, the expression of the gene fell between 1.5 and 3 times the IQR and was considered a mild outlier. However, one additional sample without a fusion product surpassed the IQR more than 3 times and was considered an extreme outlier.

In conclusion, our data suggest that the mRNA expression as detected by the array does not differ between a gene disrupted by HPV16 and the expression of that gene in samples where it has not been disrupted by the virus.

#### Viral Gene Expression

Viral gene expression could be assessed in 63 cases. APOT-PCR was able to detect a PCR product in 59 of these cases, however, the expression levels of *E2*, *E6* and *E7* as detected using RT-qPCR, varied widely. The viral gene expression of the 4 cases without detectable APOT-PCR product was nearly zero, indicating that the viral genome is not transcribed.

**Figure 4:**
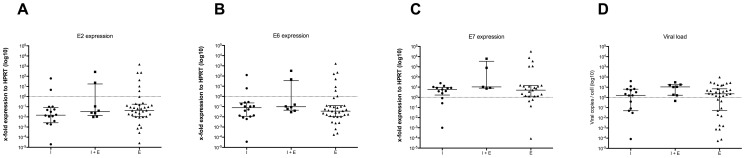
Expression of the viral genes E2, E6 and E7 and viral load. (A–C) Expression of the viral genes has been normalized to *HPRT* expression. (D) Viral load has been normalized to *β-globin*. E = episomal, E+I = episomal and integrated, I = integrated. Bars: Median with interquartile range.

When comparing cases in which a fusion transcript was detected using APOT-PCR (i.e. actively transcribed fusion product) with the remaining cases, no significant differences were seen in the mean log2 expression levels of either *E2* (1717 vs. 97; *p* = 0.308), *E6* (1859 vs. 195, *p* = 0.344) or *E7* (1724 vs. 8, *p* = 0.2943) (see Figure 4). Rather, a large variation in expression levels of these viral *E2*, *E6* and *E7* genes was observed, independent of HPV integration status.

### Viral Load

To examine whether tumors with episomal virus have a higher viral load than those with integration as determined by APOT- and/or DIPS-PCR, we have performed qPCR in 73 OSCC samples. Viral load ranged from 3.4*10^−6^ up to 97 HPV DNA copies per cell. When comparing the average viral load in cases in which a fusion product was detected using APOT- and/or DIPS-PCR with the remaining cases, no significant differences were seen (7 vs. 8.5 HPV DNA copies/cell; *p* = 0.683). No correlation was seen between the mean log2 expression levels of the viral genes *E2*, *E6* or *E7* and the viral load.

## Discussion

In this study we have comprehensively analyzed a large collection of 75 HPV16 positive OSCC for their HPV16 physical status (episomal vs. integrated) and its relation to viral oncogene expression and virally disrupted human genes. In particular we were interested to see if cases with proven integration would show higher *E6/E7* viral oncogene expression than E2 expression as suggested by studies with cervical cancer cell lines [17],[19]–[21]. By detecting viral-human fusion products with APOT- and/or DIPS-PCR in 39% of these cases we provided direct evidence for viral integration. The so-called episomal products obtained by DIPS- and/or APOT-PCR in the remaining cases are indicative for the presence of episomal HPV DNA, although they by themselves provide no proof for this assumption, because they could eventually also arise from integrated head to tail repeats of the viral genome. In this respect, two recently published studies have shown that using DIPS-PCR with other primer combinations or multiplex PCR followed by massive parallel sequencing may detect additional sites of HPV integration which is in agreement with our findings comparing DIPS- and APOT-PCR [40],[41]. The expression of HPV16 interrupted genes as well as viral genes *E2*, *E6* and *E7* in the tumors analyzed here, however, did not differ significantly from cases where no fusion product was detected. Furthermore, the cases with integration showed no notable differences in viral load in comparison with the remaining tumors. These data indicate that HPV16 integration in these tumors does not necessarily affect the levels of HPV-disrupted human gene transcripts as detected by mRNA expression arrays and/or viral gene transcripts. Thus constitutive rather than a high level of expression of oncogene transcripts appears to be required in HPV-related OSCC.

We identified integration sites by APOT- and/or DIPS-PCR in 27 out of 75 OSCC, of which 21 showed one, 5 showed two and 1 case showed four integration sites. In addition, 8 of these 27 tumors also harbored episomal viral DNA. Exclusively viral HPV16 DNA or RNA sequences indicating the presence of episomal virus were identified in the remaining 48 OSCC. This finding is in agreement with results on a series of HPV16 positive cervical squamous cell carcinomas in which 55% showed viral integration by APOT-PCR [11]. In the OSCC, integration sites showed to be distributed all over the human genome with half of them near fragile sites and some of them in previously detected clusters of viral integration (3q28, 8q24.21, 13q22.1 and 17q21) [42]. Interestingly, in 27 out of 37 detected sequences HPV16 directly interrupted known or predicted genes. Taken together, these data suggest that HPV16 integration is not simply a random event, but rather has a preference for less protected and more accessible chromosomal regions like transcribed tumor-genes and fragile sites. It can be speculated that integration takes place in genes which are highly expressed during carcinogenesis rather than that the integration itself affecting the genomic sites is the driving force. Nevertheless, another hypothesis might be that integration occurs randomly and cells with integration in particular genes preferably develop into a carcinoma. However, this is difficult to study, because premalignant lesions with HPV-infection developing in a carcinoma are seldom found.

We had access to mRNA expression profiling data of a subset of the OSCC used in this study including 6 cases with proven integration (7 sites in total). In these cases integration of HPV16 occurred within gene sequences, including the known tumor related genes *FANCC*, *HDAC2*, *SYNPO2* and *TRAF3*. Indeed, expression of *FANCC* and *HDAC2* genes has been reported to play a role in HNSCC [43]–[46]. Viral integration, however, did not lead to significantly different expression of the interrupted gene in comparison to OSCC having integration in another DNA sequence or showing solely episomal virus. This is in contrast to a recent study of our group showing that integration of low-risk HPV6 in the *AKR1C3* gene resulted in loss of gene expression in a laryngeal carcinoma [47]. In this case, however, the other gene copy was lost in the tumor as shown by array CGH analyses. In the 6 OSCCs studied here, no loss of the chromosomal regions containing the virally interrupted genes has been detected by array CGH (Olthof, Lam, unpublished results). This indicates that one or more expressed gene copies are still present in these tumors, which can mask a possible effect of the integration on gene expression. On the other hand, this might also point to the fact that viral integration is not per se meant to deregulate the interrupted gene in the cell, as also can be concluded by the finding of HPV16 integrated in intergenic sequences of 10 OSCC in this study. In conclusion, these data suggest that if there is an effect of viral integration on carcinogenesis, affecting the genomic site is unlikely to be the driving force in OSCC. Nevertheless, this has to be confirmed on the protein level in further studies.

Alternatively, integration might have an effect on viral oncogene *E6* and/or *E7* expression. In this respect it has been hypothesized that integration leads to disruption of the viral *E2* gene, which as a consequence cannot regulate *E6* and *E7* gene expression anymore from the LCR promoter region. Our DIPS-PCR data show that integration always affected the *E2* gene, either by disrupting the viral *E2* gene itself (38%) or the upstream *E1* gene (62%), also leading to *E2* loss. Nevertheless, in most of these tumors E2 mRNA transcripts were detectable at different levels of expression and these transcript levels did not differ significantly from those detected in OSCC with episomal virus. This is in contrast to the results of Häfner et al., which showed a decrease in *E5* transcript levels (downstream of *E2*) in uterine cervical lesions with integrated HPV16 [22]. Nevertheless, a rather constant transcription level of *E6* oncogene transcripts was detected independent of the physical status of the virus in these lesions. In OSCC, we also observed a broad distribution for *E6* and *E7* transcript levels independent of a detected viral integration event. This points to the fact that a constitutive expression of viral transcripts seems to be required within tumors. Only in a few cases very high levels of viral gene transcripts (*E2* as well as *E6* and *E7*) were detected, indicating that mechanisms other than *E2* binding to the viral LCR promoter region might influence transcription levels such as methylation of the LCR region [48],[49] [50].

Although more viral-cellular fusion products can be detected by using both DIPS- and APOT-PCR, a limitation of using these two assays simultaneously lies in the fact that they can result in a different outcome. For example, in two cases where we found integration sequences for both DIPS- and APOT-PCR, the viral-cellular fusion transcript sequence turned out to be in the opposite orientation as the sequence detected by DIPS-PCR. In a third case the integration sequences identified by both techniques were 20 Mb apart from each other on chromosome 22 (sample no 11, Table S1). This might be explained by previous studies showing that HPV-DNA integration can lead to both complex rearrangements changing the orientation of the 5′- and 3′ cellular sequences flanking the viral integration site, as well as amplifications and deletions of larger genomic regions starting at the viral integration site [51],[52].

In some cases (e.g. no. 14–16 and 18, Table S1) we detected integration by DIPS-PCR and episomal copies by APOT-PCR. An explanation for this finding could be a transcriptionally silent integration, for instance as a result of methylation, or if many episomal copies are present in a tumor, either in episomes or integrated in head-to-tail tandem repeats, the identification of fusion products might be difficult. We also analyzed tumors (e.g. no. 20–29, Table S1), in which a fusion product was detected by APOT-PCR, and DIPS-PCR resulted in episomal viral copies or no PCR product. This might be due to the detection of head-to-tail tandem repeats integrated into the genome or viral integration at other disruption sites of the viral genome that can not be detected by the used DIPS-PCR approach [41].

In conclusion our data indicate that HPV physical status (extrachromosomal episomes or host DNA integrated) does not affect the levels of viral and/or HPV-disrupted human gene transcripts. Therefore constitutive and not a high level of expression of oncogene transcripts appears to be required in HPV-related OSCC.

## Supporting Information

Figure S1 **Representative examples of (A) APOT-PCR and (B, C) DIPS-PCR.** (A) T60 and T65 represent tumors with episomal viral copies and T1, T2, T20 and T3 tumors with viral integration, respectively. A HPV-negative tumor sample was used as negative control. (B) Representative examples of a tumor with episomal viral copies (T60) and a tumor with integrated viral DNA (T16). A tumor sample with proven episomal viral copies was used as positive control (Primer 3 PCR is shown). (C) DIPS-PCR controls using HPV16 and HPV18 plasmid DNA. PCR fragments extracted for sequencing are marked with an asterisk. Marker: Fermentas GeneRuler DNA Ladder Mix. (TIF)

Figure S2 **Chromosomal distribution of viral integration sites.** Integration sites were found all over the genome, except for chromosomes 11, 16, 19, 20, 21 and X. Integration sites are indicated on the right side of each chromosome and fragile sites that are located within 5 MB of the nearest integration site are indicated on the left side of the chromosome. Integration sites detected by APOT-PCR are indicated by red triangles, sites detected by DIPS-PCR are indicated by blue squares and sites detected by both methods are indicated by black filled circles. (TIF)

Figure S3 **Episomal and integration derived mRNA splicing types.** Type A shows splicing from HPV E1 (nucleotide 880) to the cellular sequence. Type B shows HPV E1 spliced to HPV E4 and subsequently to the cellular DNA. Type C transcripts are not spliced (not observed in this study). Type D shows splicing from viral E1 to viral nucleotide 409 and subsequently from viral E7 to the cellular sequence. Viral DNA is indicated as such, since it has not been sequenced. (TIF)

Table S1 **Summary of HPV fusion products analyzed.** Cases where only episomal HPV16 was detected, which were spliced regularly from HPV:880∧HPV3358 are not mentioned in this table (n = 43). ^a^Indicates pathology of the primary tumor where BOT = base of tongue, O = oropharynx, PM = palatum molle and T = tonsil. ^b^HPV stat indicates (E)pisomal or (I)ntegrated status of HPV as detected by used method. ^c^Viral disruption (nt) indicates the last nucleotide of HPV sequence. ^d^Viral insertion (nt) indicates the first nucleotide of the insertion site for the human genome, where (+) indicates forward and (−) indicates reverse strand. ^e^Integration locus indicates whether integration has taken place in an intron (int), exon (ex) or intergenic region (inter) and whether in the coding or opposite (opp) strand. The intron or exon number is also indicated. ^f^GenBank gene name and accession number of corresponding whole chromosome sequence. ^g^Fragile sites according to NCBI Map View, for distances ≥5 Mb the approximate distance to viral insertion site is indicated. ^h^Transcript type, where A = splicing directly to the human sequence and B = internal splicing in HPV before splicing to the human sequence. ^i^Splice structure from viral donor site 880 (HPV880) to viral acceptor site (∧HPVnucleotide) and/or human genome as indicated (∧HSC_chromosome number:(strand)nucleotide). HSC = homo sapiens chromosome. ^j^Acceptor site indicates whether splicing has taken place to an intron (int), exon (ex) or intergenic region (inter) and whether in the coding or opposite (opp) strand. The intron or exon number is also indicated. All Data refer to GRCh37.p5 Primary Assembly. Numbering of HPV16 sequence according to GenBank Accession number NC_001526. Abbreviation: us: upstream, ds: downstream. (DOCX)
