# Supplementary figures and images for: Comprehensive Analysis of HPV16 Integration in OSCC Reveals No Significant Impact of Physical Status on Viral Oncogene and Virally Disrupted Human Gene Expression
Source: PLoS One. 2014 Feb 24;9(2):e88718. doi: 10.1371/journal.pone.0088718 (PMC3933331; doi:10.1371/journal.pone.0088718)

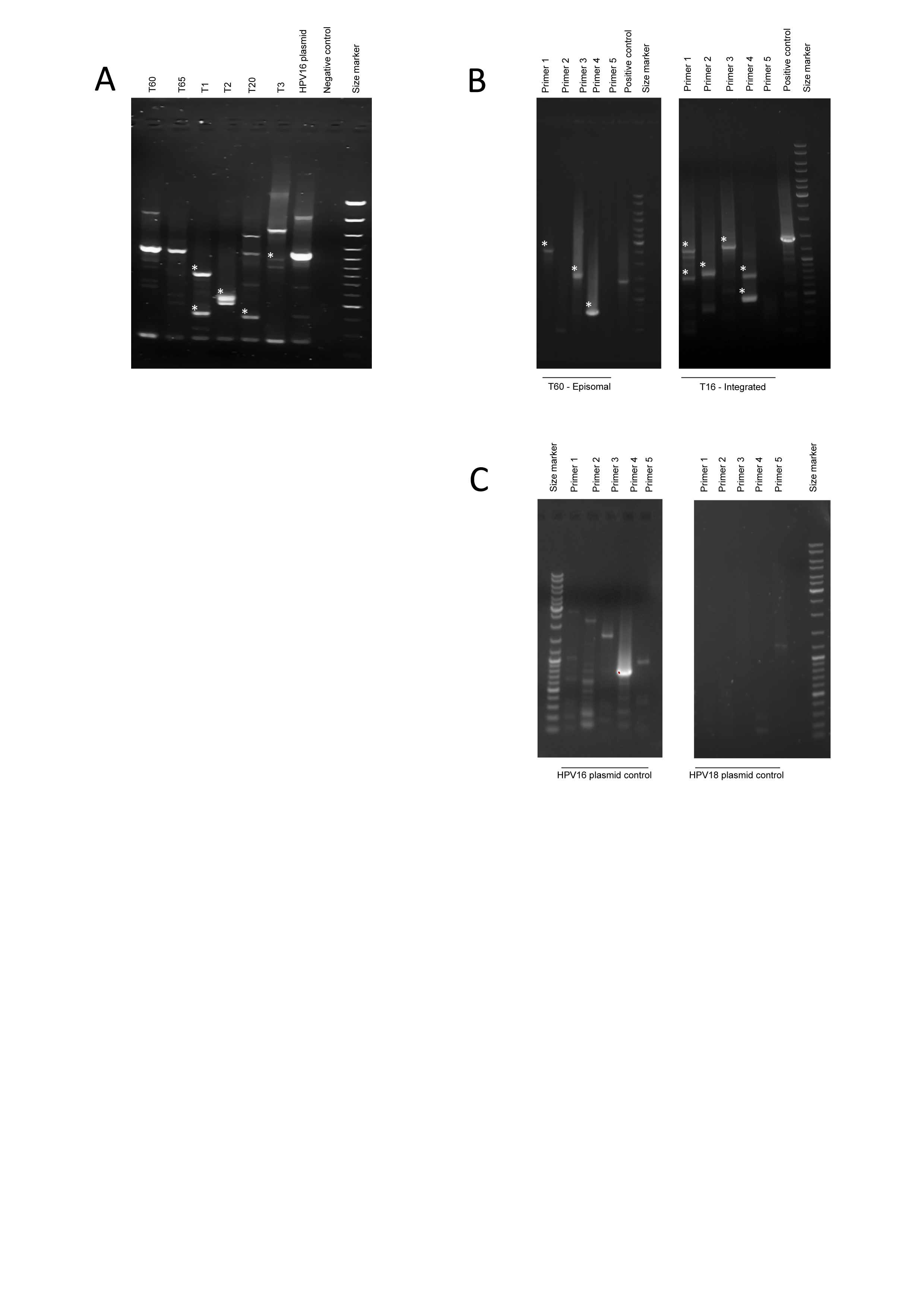

Supplement: Figure S1 — (TIF) [file pone.0088718.s001.tif]

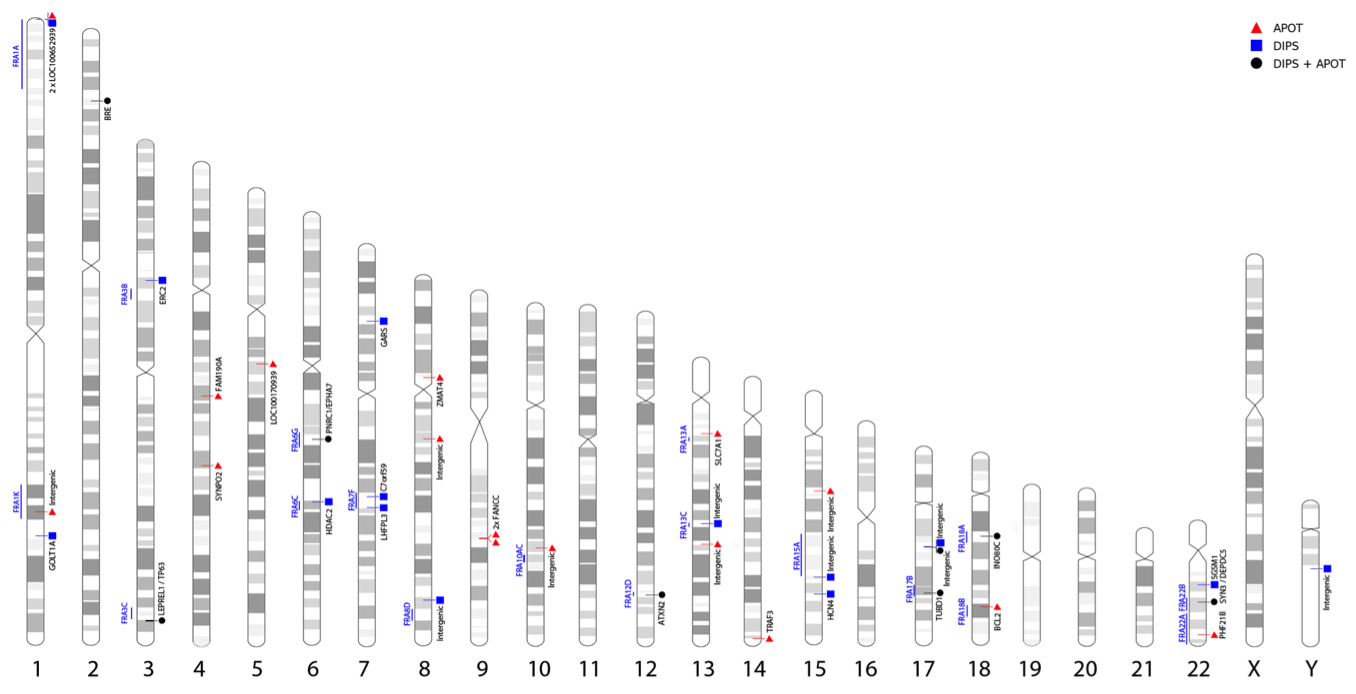

Supplement: Figure S2 — (TIF) [file pone.0088718.s002.tif]

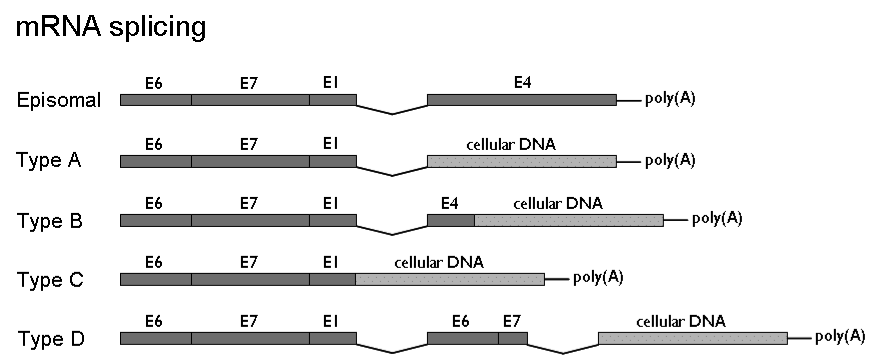

Supplement: Figure S3 — (TIF) [file pone.0088718.s003.tif]
