# Supplementary material for: Comprehensive Analysis of HPV16 Integration in OSCC Reveals No Significant Impact of Physical Status on Viral Oncogene and Virally Disrupted Human Gene Expression
Source: PLoS One. 2014 Feb 24;9(2):e88718. doi: 10.1371/journal.pone.0088718 (PMC3933331; doi:10.1371/journal.pone.0088718)
Supplement: Table S1 — (DOCX) [file pone.0088718.s004.docx]

**Table S1: Summary of HPV fusion products analyzed.**

|  |  |  | **DIPS-PCR** | | | | | | | **APOT-PCR** | | | | | | |
| --- | --- | --- | --- | --- | --- | --- | --- | --- | --- | --- | --- | --- | --- | --- | --- | --- |
| *No* | *Path.^a^* | *Sex* | *HPV stat.^b^* | *Viral disruption^c^ (nt)* | *Viral insertion^d^ (nt)* | *Map* | *Integr. locus^e^* | *Database comparison^f^* | *Fragile site^g^* | *HPV stat.^b^* | *Transcr. type^h^* | *mRNA splice structure^i^* | *Map* | *Acc.*  *site^j^* | *Database comparison^f^* | *Fragile site^g^* |
| 1 | T | M | I | 3005 (E2) | 1:(-)204168342 | 1q32.1 | Ex 4 coding | **GOLT1A** *NC_000001.10* | FRA1K  1q31  (~11 Mb) | I | A | HPV:880^HSC_  8:(-)64769670 | 8p12.3 | Inter | *NC_000008.10* | - |
|  |  |  | I | 1392 (E1) | 3:(-)55545015 | 3p14.3 | Ex 18 coding | **ERC2** *NC_000003.11* | FRA3B  3p14.2 (~5 Mb) | I | A | HPV:880^HSC_  8:(-)64812858 | 8p12.3 | Inter | *NC_000008.10* | - |
|  |  |  | I | 1366 (E1) | Y:(+)27100111 | Yq11.223 | Inter | *NC_000024.9* | - | - | - | - | - | - | - | - |
| 2 | T/PM | M | I | 2383 (E1) | 2:(-)28508231 | 2p23.2 | Intr 11 opp. | **BRE**  *NC_000002.11* | FRA2C  2p24.2 (~9 Mb) | I | A | HPV880^HSC_  2:(-)28490696 | 2p23.2 | Intr 11 opp. | **BRE**  *NC_000002.11* | FRA2C  2p24.2 (~9 Mb) |
| 3 | BOT | M | I | 2253 (E1) | 17:(-)39827047 | 17q21.2 | Inter | *NC_000017.10* | FRA17B  17q23.1 (~18 Mb) | I | A | HPV:880^HSC_  17:(-)39824881 | 17q21 | Inter | *NC_000008.10* | FRA17B  17q23.1 (~18 Mb) |
| 4 | T | F | I | 2313 (E1) | 18:(+)33076259 | 18q12.2 | Intr 1 opp. | **INO80C** *NC_000018.9* | FRA18A  18q12.2 | I | A | HPV:880^HSC_  18:(+)33079015 | 18q12.2 | opp. | **INO80C** *NC_000018.9* | FRA18A  18q12.2 |
| 5 | T | M | I | 2119 (E1) | 6:(+)94112296 | 6q16.1 | Intr 3  opp. | **EPHA7** *NC_000006.11* (PNRC1 ~4 Mb us) | FRA6G  6q15 | I | A | HPV:880^HSC_  6:(+)89790877 | 6q15 | Intr 1 coding | **PNRC1** *NC_000006.11* | FRA6G  6q15 |
|  | T | M | - | - | - | - | - | - | - | I | D | HPV:880^HPV:  409-708^HSC_  1:(+)564778 | 1p36.33 | Intr 1 coding | **LOC100652939** *NC_000001.10* | FRA1A  1p36 |
| 6 | T | F | I | 1403 (E1) | 3:(-)189782873 | 3q28 | Intr 1  coding | **LEPREL1** *NC_000003.11* (TP63 215,5 kB ds) | FRA3C  3q27  (~5,5 Mb) | I | A | HPV:880^HSC_  3:(-)189567320 | 3q28 | Intr 4 opp. | **TP63** *NC_000003.11* | FRA3C  3q27  (~5,5 Mb) |
| 7 | T | F | I | 1767 (E1) | 22:(-)33262720 | 22q12.3 | Intr 5 coding | **SYN3** *NC_000022.10* | FRA22B  22q12.3 | I | B | HPV:880^HPV:  3366-3631^HSC_  22:(+)32302191 | 22q12.3 | Ex 39 coding | **DEPDC5** *NC_000022.10* | FRA22B  22q12.3 |
| 8 | T/BOT | F | I | 3351 (E2) | 15:(-)73615951 | 15q24.1 | Ex 8 coding | **HCN4** *NC_000015.9* | FRA15A  15q22  (~13 Mb) | I | A | HPV:880^HSC_  5:(+)69451328 | 5q13 | Intr 4 opp. | **LOC100170939** *NC_000005.9* | FRA5B  5q15 (~25 Mb) |
| 9 | O | M | I | 1538 (E1) | 15:(-)80225117 | 15q25.1 | Inter | *NC_000015.9* | FRA15A  15q22  (19,5 Mb) | I | D | HPV:880^HPV:  409-718^HSC_  15:(+)39692287 | 15q15 | Inter | *NC_000015.9* | FRA15A  15q22 (~21 Mb) |
|  |  |  | - | - | - | - | - | - | - | E | - | HPV:880^HPV:3356 | - | - | - | - |
| 10 | T | M | I | 3097 (E2) | 6:(+)114267275 | 6q21 | Ex 9 opp. | **HDAC2** *NC_000006.11* | FRA6F  6q21 | I | A | HPV:880^HSC_  14:(+)103336522 | 14q32.32 | Ex 3 coding | **TRAF3**  *NC_000014.8* | FRA14C  14q24.1 |
| 11 | O/P | M | I | 1334 (E1) | 22:(-)25233023 | 22q11.23 | Intr 1  opp. | **SGSM1** *NC_000022.10* | FRA22B  22q12.3 (~6 Mb) | I | A | HPV:880^HSC_  22:(+)45346809 | 22q13.31 | Intr 3 coding | **PHF21B**  *NC_000022.10* | FRA22A  22q13 (~6,5 Mb) |
| 12 | T | M | I | 1340 (E1) | 12:(-)111918119 | 12q24.1 | Intr 18 coding | **ATXN2** *NC_000012.11* | FRA12C  12q24.2 | I | A | HPV:80^HSC_  12:(-)111908546 | 12q24.1 | Ex 18 coding | **ATXN2** *NC_000012.11* | FRA12C  12q24.2 |
| 13 | T | M | I | 3061 (E2) | 17:(+)57921102 | 17q23.1 | Inter | *NC_000017.10* (TUBD1 16 kB ds) | FRA17B  17q23.1 | I | A | HPV:880^HSC_  17 :(+)57947254 | 17q23.1 | Ex 3 opp. | **TUBD1** *NC_000017.10* | FRA17B  17q23.1 |
| *(continued on next page)* | | | | | | | | | | | | | | | | |

| *(Table 1 continued)* | | | | | | | | | | | | | | | | |
| --- | --- | --- | --- | --- | --- | --- | --- | --- | --- | --- | --- | --- | --- | --- | --- | --- |
| 14 | T | M | I + E | 2283 (E1) | 7:(+)30661954 | 7p15 | Ex 12 coding | **GARS**  *NC_000007.13* | FRA7C  7p14.2 (~6 Mb) | E | - | HPV:880^HPV:3358 | - | - | - | - |
| 15 |  | M | I | 3283 (E2) | 13:(+)65063287 | 13q21 | Inter | *NC_000013.10* | FRA13C  13q21.2 | E | - | HPV:880^HPV:3358 | - | - | - | - |
| 16 | T | M | I + E | 2875 (E2) | 17:(+)39678949 | 17q21.2 | Inter | *NC_000017.10* | FRA17B  17q23.1  (~18 Mb) | E | - | HPV:880^HPV:3358 | - | - | - | - |
| 17 | T/BOT | M | I | 1124 (E1) | 7:(-)99750064 | 7q21.1 | Intr 2  opp. | **C7orf59** *NC_000007.13* | FRA7F  7q22 | ndb | - | - | - | - | - | - |
| 18 | T | M | I | 2894 (E2) | 8:(-)128266756 | 8q24.21 | Inter | *NC_000008.10* | FRA8D  8q24.3 | E | - | HPV:880^HPV:3358 | - | - | - | - |
| 19 | O | M | I | 2908 (E2) | 7:(-)104159073 | 7q22.2 | Intr 1  opp. | **LHFPL3** *NC_000007.13* | FRA7F  7q22 | ndb | - | - | - | - | - | - |
| 20 | T | M | E | - | - | - | - | - | - | I | A | HPV:880^HSC_  9:(-)97869797 | 9q22.3 | Intr 13 coding | **FANCC**  *NC_000009.11* | FRA9D  9q22.1 (~6 Mb) |
| 21 | T | F | ndb | - | - | - | - | - | - | I | A | HPV:880^HSC_  18:(-)60840487 | 18q21.3 | Intr 2 coding | **BCL2** *NC_000018.9* | FRA18B  18q21.3 |
| 22 | BOT | F | ndb | - | - | - | - | - | - | I | D | HPV:880^HPV:  409-720^HSC_  1(+)564788 | 1p36.33 | Intr 1 coding | **LOC100652939** *NC_000001.10* | FRA1A  1p36 |
| 23 | T | M | E | - | - | - | - | - | - | I | D | HPV:880^HPV:  409-708^HSC_  HSC_10:(-)95015832 | 10q23.33 | Inter | *NC_000010.10* | FRA10AC1  10q23.3 |
| 24 | T | F | ndb | - | - | - | - | - | - | I | A | HPV:880^HSC_  9:(-)97947013 | 9q22.3 | Intr 4  coding | **FANCC** *NC_000009.11* | FRA9D  9q22.1 (~6 Mb) |
| 25 | T | M | ndb | - | - | - | - | - | - | I | A | HPV:880^HSC_  13:(+)73910828 | 13q22 | Inter | *NC_000013.10* | FRA13C  13q21.2 (~12 Mb) |
| 26 | T | M | - | - | - | - | - | - | - | I | A | HPV:880^HSC_  13:(+)30135781 | 13q12 | Ex 1 opp. | **SLC7A1** *NC_000013.10* | FRA13A  13q13.3 (~4 Mb) |
| 27 | O | M | E | - | - | - | - | - | - | I | A | HPV:880^HSC_  1:(-)194643196 | 1q31 | Inter | *NC_000001.10* | FRA1K  1q31 |
|  |  |  | - | - | - | - | - | - | - | I | A | HPV:880°HSC_  4:(+)92436058 | 4q22.1 | Intr 9  coding | **FAM190A**  *NC_000004.11* | - |
| 28 | T | M | E | - | - | - | - | - | - | I | A | HPV:880^HSC_  8:(+)40561379 | 8p11.21 | Ex 3 opp. | **ZMAT4**  *NC_000008.10* | - |
| 29 | T | M | - | - | - | - | - | - | - | I | A | HPV:880^HSC_  4:(-)119883351 | 4q26 | Ex 1  opp. | **SYNPO2**  *NC_000004.11* | - |
| 30 | T | M | E | - | - | - | - | - | - | E | - | HPV:880^HPV:409 | - | - | - | - |
| 31 | T | M | E | - | - | - | - | - | - | E | - | HPV:880^HPV:2702 | - | - | - | - |
| 32 | T | M | E | - | - | - | - | - | - | E | - | HPV:880^HPV:2707 | - | - | - | - |

Cases where only episomal HPV16 was detected, which were spliced regularly from HPV:880^HPV3358 are not mentioned in this table (n=43). ^a^Indicates pathology of the primary tumor where BOT= base of tongue, O=oropharynx, PM=palatum molle and T=tonsil. ^b^HPV stat indicates (E)pisomal or (I)ntegrated status of HPV as detected by used method. ^c^Viral disruption (nt) indicates the last nucleotide of HPV sequence. ^d^Viral insertion (nt) indicates the first nucleotide of the insertion site for the human genome, where (+) indicates forward and (-) indicates reverse strand. ^e^Integration locus indicates whether integration has taken place in an intron (int), exon (ex) or intergenic region (inter) and whether in the coding or opposite (opp) strand. The intron or exon number is also indicated. ^f^GenBank gene name and accession number of corresponding whole chromosome sequence. ^g^Fragile sites according to NCBI Map View, for distances ≥5 Mb the approximate distance to viral insertion site is indicated. ^h^Transcript type, where A=splicing directly to the human sequence and B=internal splicing in HPV before splicing to the human sequence. ^i^Splice structure from viral donor site 880 (HPV880) to viral acceptor site (^HPVnucleotide) and/or human genome as indicated (^HSC_chromosome number:(strand)nucleotide). HSC = homo sapiens chromosome. ^j^Acceptor site indicates whether splicing has taken place to an intron (int), exon (ex) or intergenic region (inter) and whether in the coding or opposite (opp) strand. The intron or exon number is also indicated. All Data refer to GRCh37.p5 Primary Assembly. Numbering of HPV16 sequence according to GenBank Accession number NC_001526. Abbreviation: us: upstream, ds: downstream
